# Efficacy and tolerability of DAAs in HCV-monoinfected and HCV/HIV-coinfected patients with psychiatric disorders

**DOI:** 10.1186/s12879-020-4922-2

**Published:** 2020-03-06

**Authors:** Nicolò de Gennaro, Lucia Diella, Laura Monno, Gioacchino Angarano, Michele Milella, Annalisa Saracino

**Affiliations:** Clinic of Infectious Diseases, University of Bari, University Hospital Policlinico, Piazza Giulio Cesare n. 11, 70124 Bari, Italy

**Keywords:** HCV, Antiviral treatment, SVR, Psychiatric comorbidity

## Abstract

**Background:**

Few data are available regarding the use of direct antiviral agents (DAAs) for chronic hepatitis C in psychiatric patients. The aim of the study is to assess safety and outcome of DAAs in patients with psychiatric comorbidities.

**Methods:**

This retrospective, observational, single-centre study enrolled patients treated with psychiatric drugs who initiated DAAs between 2015 and 2018. Patients were classified into two groups: A (on anxiolitycs/antidepressant) and B (on antipsychotics). Week-12 sustained virological response (SVR-12) and adverse events (AEs) were evaluated.

**Results:**

One hundred forty-four patients were included (A:101; B:43). Patients were 49.3% males, mean age 60 years (SD ± 13.5); 31.9% cirrhotic; 125 (86.8%) HCV-monoinfected and 19 (13.2%) HCV /HIV-coinfected. Twenty patients (13.8%) required a change of psychiatric therapy before initiation of DAA. Overall, SVR-12 was achieved in 88.2% of subjects in intention-to-treat(ITT)-analysis. Lower SVR rates were observed in group B vs A (79% vs 92%, *p* = 0.045) and in those changing psychiatric drugs vs others (8% vs 30%, *p* = 0.015). According to per-protocol (PP)-analysis, SVR-12 was achieved in 93/95 (97.9%) in group A versus 34/36 (94.4%) in group B (*p* = 0.30). At least one AE occurred in 60 patients (41.6%), including 10 severe AEs, leading to 3 discontinuations. AEs were more frequently reported in group A (*p* = 0.015).

**Conclusions:**

The study confirms effectiveness and safety of DAA-based treatment also in this special population, even if a careful evaluation of history and drug-drug interactions is warranted.

## Background

Nowadays, it is estimated that 70 million persons are living with hepatitis C virus (HCV) worldwide, including 1% of the entire global population according to recent estimates [1]. In addition to the liver-related mortality and morbidity, HCV-infection represents as a systemic disease, as HCV shows a considerable tropism for other tissues and organs [2]. Specifically, HCV-infection has been associated with several extrahepatic manifestations among which neuropsychiatric disorders are described in up to half 50% of chronic HCV-infected patients [3, 4]. Psychiatric symptoms such as cognitive impairment, asthenia, weakness, anxiety and depression have been reported with high frequency in patients with chronic HCV-infection, causing interference with patient ability to perform daily activities and worsening quality of life [5]. This evidence of neurocognitive impairment in some patients with HCV-infection, is not fully attributable to liver dysfunction. Although many researches have suggested a pivotal pathogenetic role of HCV in the Central Nervous System (CNS) [6, 7] to explain the high rate of psychiatric comorbidity among these patients, probably the adoption of risk behaviors is the main cause of HCV-infection [8].

Patients with such comorbidity have often been excluded from treatment with Interferon (IFN)-based regimens because of the elevated risk of an exacerbation of psychiatric symptoms [9]. In addition, studies indicated psychiatric comorbidity as a risk factor for non-adherence and not achievement of sustained virological response (SVR) [10]. The recent introduction of IFN-free therapeutic regimens based on direct-acting antiviral agents (DAAs) has revolutionized HCV therapy and viral cure associated with improved quality of life is now a reality in the vast majority of patients. The last therapeutic regimens approved by Food and Drug Administration (FDA) and European Medicines Agency (EMA) are pangenotypic, once-daily, all-oral DAA combinations that have the potential to close the gaps in the current DAA treatment portfolio. Eight-twelve weeks of treatment is now the standard of care and viral eradication can be achieved in > 95% across different patient populations [11]. However, limited information is currently available regarding the use of DAAs in subjects with psychiatric comorbidity [12–16].

Moreover, the choice of the best correct therapeutic DAA-regimen in these patients is complicated by drug-drug interactions (DDIs) with any psychiatric drug [11].

Therefore, this study seeks to evaluate the efficacy and tolerability of DAA-based antiviral therapy among HCV-monoinfected and HCV/HIV-coinfected patients with psychiatric comorbidity.

## Methods

All consecutive HCV-infected patients (≥18 years old) presenting a documented psychiatric comorbidity, with or without HIV infection, who initiated a DAA-based regimen between February 2015 and June 2018 at the Infectious Diseases Clinic, University of Bari, were included in this retrospective, observational study. Patients with a documented psychiatric comorbidity were defined as subjects who had received a previous diagnosis by a psychiatric specialist and for whom a psychiatric drug had been initiated. For purposes of analysis, patients were divided based on their psychiatric treatment into two groups: subjects experienced with anxiolytic and/or antidepressant (group A) and subjects on treatment with antipsychotic (group B).

Demographic, clinical and biochemical data, including type of psychiatric illness and concomitant co-medications at baseline were registered for all subjects.

Potential DDIs were evaluated before the administration of DAAs using a web interaction-checker (available at www.hepdruginteractions.org) and changes in co-medications due to DDIs were also recorded. During treatment, all patients routinely underwent monthly monitoring, including clinical and laboratory assessment.

Efficacy assessment (primary endpoint: SVR-12) was based on an intention-to-treat (ITT) analysis, therefore all patients who received at least one dose of anti-HCV medication were included. Moreover, a per protocol (PP)-analysis was performed excluding non compliant and lost to follow-up patients. Non-compliance was evaluated by physicians considering a reported history of incomplete or irregular drug intake.

Safety profile of DAAs (secondary endpoint) was evaluated during each visit in all patients by a dedicated medical equipe, by means of focused questions regarding any sign and/or symptom occurred during treatment. The number of AEs per person was recorded, as well as their type and grade of severity.

### DAA-based anti-HCV treatment

The following regimens were administered based on international recommendations [17]: sofosbuvir (SOF) plus ribavirin (RBV); SOF plus simeprevir (SMV) ± RBV; SOF plus ledipasvir (LDV) ± RBV; SOF plus daclatasvir (DCV) ± RBV; ombitasvir (OBV), paritaprevir/ritonavir (PTV/r) ± dasabuvir(DSV) ± RBV, glecaprevir (GLE) plus pibrentasvir (PIB), grazoprevir (GRZ) plus elbasvir (EBR) ± RBV, SOF plus velpatasvir (VEL) ± RBV. Use of RBV was based on clinical judgement and on the prescribing information document; in particular, an initial dose of 1000 mg or 1200 mg was prescribed according to the patients’ weight (< 75 kg or ≥ 75 kg, respectively).

### HCV-RNA measurement and HCV genotype assessment

Plasma HCV-RNA levels were measured for all patients at baseline, at the end of treatment (EOT), and 12 weeks after EOT (SVR-12), using the Siemens Real Time PCR assay (Siemens Healthcare Diagnostics, Tarrytown, NY, USA), with a lower limit of detection of 15 IU/ml. HCV genotype and subtype were determined using the Siemens Versant HCV LiPA V2 assay (Siemens, Munich, Germany).

### Definitions

Sustained virological response was defined as an undetectable HCV-RNA level at week 12 after EOT. Virological breakthrough was defined as an undetectable HCV-RNA during treatment followed by a detectable HCV-RNA, despite continued treatment. Relapse was defined as undetectable HCV-RNA at EOT but detectable HCV-RNA during follow-up.

### Severe adverse events (SAEs)

Severe adverse events were classified according to a recent definition (Common Terminology Criteria for adverse events-CTCAE, 2017).

### Statistical analysis

Descriptive statistics were calculated for demographic, clinical, and laboratory characteristics of cases. Mean and standard deviation (SD) were recorded for normally distributed variables, and the median and interquartile range (IQR) for non-normally distributed variables. The number and percentage were recorded for categorical variables. Differences between groups were analyzed using the Fisher’s exact test, or Mann–Whitney test, as appropriate. A *p*-value of < 0.05 was considered to indicate significance. Epi Info computer software version 7 was used for statistical analysis.

### Ethics

This research did not require formal approval from the ethics committee according to Italian law, since it was performed in the context of normal clinical routines. However, the study was conducted in accordance with the Declaration of Helsinki and national and institutional standards. All patients provided a written informed consent at the time of first visit at our Centre for the use of their data for research purposes.

## Results

### Clinical-demographical features of the study population

**Fig. 1:**
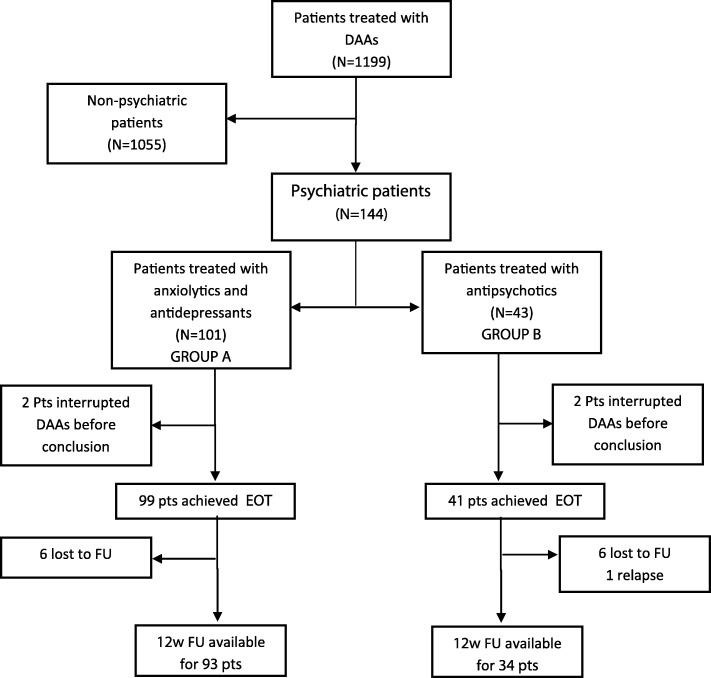
Flow-chart of pts. enrolled and evaluated from baseline to 12 weeks of follow-up

A total of 1199 HCV-infected patients (1081 HCV-monoinfected and 118 HCV/HIV-coinfected) initiated DAAs during the study period. A total of 144/1199 subjects (12.0%) presented psychiatric comorbidities and were included in the study, of whom 101 (70.1%) were on anxiolytic/antidepressant therapy (group A) and the remaining 43 (29.9%) were treated with antipsychotic drugs (group B). In Fig. 1 is represented the study diagram.

The clinical characteristics of patients at baseline are summarized in Table 1.

**Table 1:** Baseline features of the study population

	Patients *(N = 144)*	Group A *(N = 101)*	Group B *(N = 43)*	***p-value***
**Sex, males.*n*(%)**	71 (49.3%)	42 (41.6%)	29 (67.4%)	**0.006**
**Age, years, mean (SD)**	60 ± 13,5	64.9 ± 11.7	50.6 ± 12.1	**< 0.001**
**Risk Factors*n*(%)**				
*IDU*	37 (25.7%)	17 (16.9%)	20 (46.5%)	**< 0.001**
*Heterosexual*	10 (7.0%)	8 (7.9%)	2 (4.7%)	0.720
*MSM*	1 (0.7%)	0	1 (2.3%)	0.290
Nosocomial	38 (26.4%)	31 (30.6%)	7 (16.3%)	0.150
*Unknown*	58 (40.2%)	45 (44.6%)	13 (30.2%)	0.138
**HCV Genotypes*n*(%)**				
*1a*	32 (22.2%)	17 (16.8%)	15 (34.9%)	**0.020**
*1b*	51 (35.4%)	44 (43.6%)	7 (16.3%)	**0.002**
*2*	37 (25.7%)	31 (30.7%)	6 (13.9%)	**0.038**
*3*	18 (12.5%)	5 (4.9%)	13 (30.2%)	**< 0.001**
*4*	6 (4.2%)	4 (4%)	2 (4.7%)	1.000
**Duration of HCV infection, years (range)**	18 (10–25.5)	20 (11–26)	17 (9–23)	0.199
**Log 10 HCV-RNA, IU/mL, median (range)**	6.00 (5.48–6.52)	5.9 (5.6–6.5)	6.2 (5.6–6.6)	0.095
**Liver stiffness, Kpa, mean (SD)**	10,8 ± 9,9	11.5 ± 11.2	9.1 ± 5.2	0.518
**Previous failure,*n*(%)**	32 (22.2%)	29 (28.7%)	3 (7.0%)	**0.004**
**INF-based tx**	31	28	3	
**DAAs-based tx**	1	1	0	
**Fibrosis stage,*n*(%)**				
*F0-F2*	78 (54.1%)	51 (50.5%)	27 (62.8%)	0.203
*F3*	24 (16.7%)	19 (18.8%)	5 (11.6%)	0.338
*F4*	42 (29.2%)	31 (30.7%)	11 (25.6%)	0.689
**FIB-4 score median (range)**	2.02 (1.40–3.35)	2.2 (1.5–3.4)	1.6 (1.2–2.9)	**0.009**
**APRI score median (range)**	0.40 (0.60–1.22)	0.6 (0.4–1.2)	0.7 (0.4–1.2)	0.315
**MELD score median (range)**	7 (6.5–8.5)	7.0 (6.0–8.0)	7.0 (6.0–7-0)	0.923
**Child-Pugh class*n*(%)**				
A	142 (98.6%)	100 (99.1%)	42 (97.7%)	0.510
B	2 (1.4%)	1 (0.99%)	1 (2.3%)	0.510
**Cirrhosis,*n*(%)**	46 (31.9%)	34 (33.66%)	12 (27.9%)	0.562
**Oesophageal varices or portal hypertension,*n*(%)**	15 (10.4%)	11 (10.89%)	4 (9.3%)	1.000
**Type of HCV therapy*n*(%)**				
SOF + RBV	7 (4.8%)	6 (5.9%)	1 (2.3%)	0.670
SOF + SMV ± RBV	5 (3.5%)	4 (3.9%)	1 (2.3%)	1.000
SOF + LDV ± RBV	21 (14.6%)	15 (14.9%)	6 (13.9%)	1.000
SOF + DCV ± RBV	13 (9.0%)	10 (10%)	3 (7%)	0.750
OMB + PTV/r + DAS ± RBV	15 (10.4%)	15 (14.9%)	0	**0.006**
OMB + PTV/r + RBV	2 (1.4%)	2 (1.9%)	0	1.000
SOF + VEL ± RBV	37 (25.7%)	15 (14.9%)	22 (51.2%)	**< 0.001**
GLE + PIB	24 (16.7%)	17 (16.8%)	7 (16.3%)	1.000
GRZ + EBR ± RBV	20 (13.9%)	17 (16.8%)	3 (7%)	0.186
**Duration of therapy*n*(%)**				
*8w*	20 (13.9%)	15 (14.9%)	5 (11.6%)	0.794
*12w*	101 (70.1%)	67 (66.3%)	34 (79.1%)	0.164
*16w*	6 (4.2%)	4 (3.9%)	2 (4.65%)	1.000
*24w*	17 (11.8%)	15 (14.9%)	2 (4.65%)	0.097
**Addition of RBV*n*(%)**	33 (22.9%)	24 (23.74%)	9 (20.9%)	0.829
**ALT, UI/L, median (range)**	50 (30–80)	47 (26.5–78.5)	58 (43–93)	**0.029**
**Total bilirubin, mg/dL, median (range)**	0.62 (0.45–0.85)	0.65 (0.47–0.82)	0.60 (0.40–0.87)	0.323
**Serum creatinine, mg/dL, median (range)**	0.78 (0.61–0.93)	0.78 (0.60–0.95)	0.78 (0.62–0.90)	0.795
**Platelets (×10^9/L), median (range)**	183.5 (140.5–217.5)	184 (145–219)	182 (137–214)	0.936
**Albumin, mg/dL, median (range)**	4.0 (3.7–4.2)	4.0 (4.7–4.2)	4.0 (3.8–4.1)	0.976
**Body mass index, median (range)**	25.3 (23.3–28.7)	25.5 (23.8–29.1)	25.1 (22.4–27.4)	0.936
**Patients with at least 1 other comorbidity, n (%)**	101 (70.10%)	79 (78.2%)	22 (51.2%)	**0.002**
**Diabetes,*n*(%)**	20 (13.9%)	16 (15.8%)	4 (9.3%)	0.431
**Hypertension,*n*(%)**	61 (42.4%)	53 (52.5%)	8 (18.6%)	**< 0.001**
**HBsAg positive,*n*(%)**	2 (1.4%)	0	2 (4.7%)	0.087
**HIV infected,*n*(%)**	19 (13.2%)	9 (8.9%)	10 (23.3%)	**0.030**
*N aviremic pts on ART, n (%)*	19 (100%)	9 (100%)	10 (100%)	1.000
*CD4*^*+*^*(cells/μl) median (range)*	85 (42–171)	98 (50–171)	83.5 (42–186.5)	0.903
*CDC-C Stage n (%)*	7 (4.8%)	4 (3.9%)	3 (7%)	0.650

*Legend*: *IDU* Injecting drug user, *MSM* Man who have sex with man, *INF* Interferon, *DAA* Direct antiviral agent, *SOF* Sofosbuvir, *RBV* Ribavirin, *SMV* Simeprevir, *LDV* Ledipasvir, *DCV* Daclatasvir, *OMB* Ombitasvir, *PTV/r* Paripatrevir/ritonavir, *DSV* Dasabuvir, *VEL* Velpatasvir, *GLE* Glecaprevir, *PIB* Pibrentasvir, *GRZ* Grazoprevir, *EBR* Elbasvir, *ALT* Alanine aminotransferase, *ART* Antiretroviral therapy

Patients were 49.3% males with a mean age of 60 years (SD ±13.5), and 31.9% of them were cirrhotic. Patients in treatment with antipsychotic drugs were younger (50.6 vs. 64.9 years, *p* < 0.001), mostly males (67.4% vs. 41.6%, *p* = 0.006), the main route of transmission was intravenous drug use (46.5% vs. 16.9%, *p* < 0.001) and were more frequently infected with HCV genotype 1a (34.9% vs. 16.8%, *p* = 0.020), and genotype 3 (30.2% vs. 4.9%, *p* < 0.001).

Most patients (70.1% of the study population) had at least one comorbidity (other than psychiatric). Group A individuals were more likely to have at least one additional comorbidity before starting DAA-treatment (78.2% vs. 51.2%, *p* = 0.002). Arterial hypertension was the most common reported comorbidity (42.4%), mainly diagnosed in patients belonging to group A (52.5% vs. 18.6%, *p* < 0.001).

Failure to a previous anti-HCV treatment was reported in 32 patients (22.2%), the majority among patients in treatment with anxiolitycs and antidepressant (28.7% vs. 7.0%, *p* = 0.004). Among the HCV treatment-experienced subjects, one had experienced failure with a previous DAA-based regimen.

All patients had compensated liver disease according to the Child–Pugh classification. Group A subjects showed a higher FIB-4 score (2.2 vs. 1.6, *p* = 0.009). No differences in liver stiffness and fibrosis stage distribution, APRI/MELD score, and HCV-RNA at baseline were observed between the two groups.

Furthermore, 125 (86.8%) were HCV-monoinfected and 19 (13.2%) were HCV/HIV-coinfected. Coinfected patients were mostly represented among subjects in treatment with antipsychotic drugs (23.3% vs. 8.9%, *p* = 0.030). Two patients had HBsAg positivity, both in Group B.

DAA-regimens were chosen by treating clinicians based on clinical criteria and viral genotype. The most frequently prescribed DAA-regimen was SOF + VEL ± RBV (25.7% overall), especially in subjects in treatment with antipsychotic (51.2% vs. 14.9%, *p* < 0.001). The regimen including OMB + PTV/r ± DAS ± RBV was never prescribed in group B patients (*p* = 0.006).

In Table 2 the characteristics of the psychiatric disorder are detailed for both groups. Subjects belonging to group B, which included a higher percentage of former drug users, were mostly on substitution treatment with opioids at the time of inclusion in the study. The psychiatric illness’ duration was longer in group B (*p* = 0.011). No statistically significant differences between the two groups were found regarding the referred alcohol consumption.

**Table 2:** Clinical characteristics concerning psychiatric disorders at baseline

	Patients *(N = 144)*	Group A	Group B	***p***-value
**Types of psychiatric illnesses,*n*(%)**		*101 (70.1)*	*43 (29.9%)*	
*Anxiety disorders*^a^		*48 (33.3%)*		
*Mood disorders*^b^		*53 (36.8%)*		
*Psychotic disorder*			*43 (29.9%)*	
**Duration psychiatric illness, years (range)**	9 (6–13)	8 (6.5–12.5)	12 (8–15.5)	**0.011**
**Suicide attempted,*n*(%)**	5 (3.5%)	2 (2%)	3 (7%)	0.157
**Psychiatric treatment modification before DAA treatment,*n*(%)**	20 (13.9%)	11 (10.9%)	9 (21%)	0.121
**Opioid substitution treatment,*n*(%)**	12 (8.3%)	2 (2%)	10 (23.3%)	**< 0.001**
*Methadone*	11 (7.6%)	1 (1%)	10 (23.3%)	**< 0.001**
*Buprenorphine*	1 (0.7%)	1 (1%)	0	1.000
**Referred alcohol consumption,*n*(%)**	5 (3.5%)	4 (4%)	1 (2.3%)	1.000

*Legend:*^a^-social phobia-posttraumatic stress disorder-panic disorder-generalized anxiety disorder;

^b^-current major depressive episode-current manic episode-current hypomanic episode;

### Change of psychiatric and antiretroviral treatment before DAAs-initiation

Among the entire study population, a total of 20 patients (13.8%) required a modification of psychiatric therapy based on DDIs in according to the psychiatrist’ judgment before the beginning of the DAA-regimen: a dose adjustment of psychiatric drugs was observed in two patients whereas in the remaining 18 patients (10 in group A 9.9% and 8 in group B, 18.6%, *p* = 0.172) a complete therapeutic change was adopted. In seven cases (7/20, 35%) a therapeutic change was mandatory due to drug-drug interactions.

Regimens more likely to require a change of psychiatric drugs before starting DAA-treatment were SOF + DCV ± RBV (3/13 patients, 23.1%); GRZ + EBR ± RBV (4/20, 20%), SOF + VEL ± RBV (7/37, 18.9%), and GLE + PIB (3/24, 12.5%).

Among the 19 HIV/HCV-coinfected individuals, all patients were on ART at the initiation of DAA-treatment and were aviremic. A total of 6/19 patients (31.6%) required a change of ART because of DDIs, of whom four patients from group A and two from group B (*p* = 0.349). Before the ART change, 5/6 (83.3%) patients were in treatment with protease inhibitors: one with atazanavir unboosted, two with lopinavir/ritonavir, two with darunavir/ritonavir. Of the 6 patients who changed ART, only one returned to his previous ART regimen after the end of DAA-treatment. No virological failure for HIV was observed.

### Outcome

Among the 144 patients enrolled, 140/144 (97.2%) completed the prescribed DAA-regimen accomplishing EOT, while four patients prematurely interrupted their therapy: in one case due to imprisonment, and in the remaining three cases due to the occurrence of AEs (see the following paragraph).

Overall, according to an ITT-analysis, SVR-12 was achieved in 127/144 subjects (88.2%) of the study population, 93/101 (92.1%) in group A versus 34/43 (79.1%) in group B, respectively (*p* = 0.045). Clinical characteristics of psychiatric patients stratified by achieving SVR-12 are detailed in Table 3.

**Table 3:** Clinical characteristics of psychiatric patients stratified by achieving SVR-12

	SVR-12 *(N = 127)*	No SVR-12 *(N = 17)*	***p-value***
**Sex, males.*n*(%)**	58 (45.7%)	13 (76.5%)	**0.020**
**Age, years, mean (SD)**	61.8 ± 13.3	51.6 ± 11.7	**0.038**
**Risk Factors*n*(%)**			
*IDU*	28 (22.0%)	9 (52.9%)	**0.014**
*Heterosexual*	10 (7.9%)	0	0.607
*MSM*	1 (0.8%)	0	1.000
Nosocomial	36 (28.3%)	2 (11.8%)	0.239
*Unknown*	52 (41.0%)	6 (35.3%)	0.794
**HCV Genotypes*n*(%)**			
*1a*	27 (21.3%)	5 (29.4%)	0.534
*1b*	45 (35.4%)	6 (35.3%)	1.000
*2*	35 (27.6%)	2 (11.8%)	0.238
*3*	15 (11.8%)	3 (17.6%)	0.448
*4*	5 (3.9%)	1 (5.9%)	0.536
**Log 10 HCV-RNA, IU/mL, median (range)**	6.00 (5.5–6.5)	6.1 (5.5–6.7)	0.841
**Liver stiffness, Kpa, mean (SD)**	11.1 ± 10.4	8.9 ± 3.4	
**Previous failure,*n*(%)**	24 (18.9%)	8 (47.0%)	**0.024**
**INF-based tx**	23	8	
**DAAs-based tx**	1	0	
**FIB-4 score median (range)**	2.0 (1.4–3.3)	1.5 (1.3–2.1)	0.226
**APRI score median (range)**	0.6 (0.4–1.2)	0.6 (0.5–1.2)	0.936
**MELD score median (range)**	7.0 (6.0–8.0)	7.0 (6.0–7.0)	0.794
**Cirrhosis,*n*(%)**	40 (31.5%)	6 (35.3%)	0.785
**Oesophageal varices or portal hypertension,*n*(%)**	13 (10.2%)	2 (11.8%)	0.691
**Type of HCV therapy*n*(%)**			
SOF + RBV	7 (5.5%)	0	0.594
SOF + SMV ± RBV	5 (3.9%)	0	1.000
SOF + LDV ± RBV	18 (14.2%)	3 (17.6%)	0.715
SOF + DCV ± RBV	11 (8.7%)	2 (11.8%)	0.652
OMB + PTV/r + DAS ± RBV	13 (10.2%)	2 (11.8%)	0.691
OMB + PTV/r + RBV	1 (0.8%)	1 (5.9%)	0.222
SOF + VEL ± RBV	30 (23.6%)	7 (41.2%)	0.141
GLE + PIB	24 (18.9%)	0	0.076
GRZ + EBR ± RBV	18 (14.2%)	2 (11.8%)	1.000
**Addition of RBV*n*(%)**	27 (21.2%)	6 (35.3%)	0.222
**ALT, UI/L, median (range)**	50 (28.5–77.5)	70 (47–125)	**0.031**
**Total bilirubin, mg/dL, median (range)**	0.62 (0.44–0.83)	0.60 (0.40–0.80)	0.561
**Serum creatinine, mg/dL, median (range)**	0.79 (0.61–0.95)	0.78 (0.63–0.80)	0.928
**Platelets (×10^9/L), median (range)**	182 (139–219)	185 (157–210)	0.920
**Patients with at least 1 other comorbidity, n (%)**	94 (74%)	7 (41.2%)	**0.009**
**Diabetes,*n*(%)**	18 (14.2%)	2 (11.8%)	1.000
**Hypertension,*n*(%)**	57 (44.9%)	4 (23.5%)	0.119
**HIV infected,*n*(%)**	17 (13.4%)	2 (11.8%)	1.000

*Legend*: *IDU* Injecting drug user, *MSM* Man who have sex with man, *INF* Interferon, *DAA* Direct antiviral agent, *SOF* Sofosbuvir, *RBV* Ribavirin, *SMV* Simeprevir, *LDV* Ledipasvir, *DCV* Daclatasvir, *OMB* Ombitasvir, *PTV/r* Paripatrevir/ritonavir, *DSV* Dasabuvir, *VEL* Velpatasvir, *GLE* Glecaprevir, *PIB* Pibrentasvir, *GRZ* Grazoprevir, *EBR* Elbasvir, *ALT* Alanine aminotransferase, *ART* Antiretroviral therapy

Among the patients achieving EOT but not SVR-12, twelve were lost to follow-up (6 from group A and 6 from group B), and only one (belonging to group B) had a relapse. This patient was a non-cirrhotic subject infected with HCV genotype 3, who had received the SOF + DCV + RBV combination for 12 weeks; he had resistance testing performed at failure which demonstrated a resistance pattern against NS5A (Y93H).

A lower SVR rate (79%), was observed in psychotic patients (group B) compared to group A (92%) which was mostly due to a higher prevalence of patients lost to follow-up (6/43, 14% vs. 6/101, 6% respectively, *p* = 0.183).

Patients who did not achieve SVR-12 were mostly males (76.5% vs 45.7% in responders patient, *p* = 0.020), younger (51.6 vs 61.8, *p* = 0.038), more frequently intravenous drug users (52.9% vs 22%, *p* = 0.014) and presented a higher rate of failure to a previous anti-HCV treatment (47.0% vs 18.9%, *p* = 0.024). The proportion of patients not achieving SVR-12 was higher among patients who underwent a change of the psychiatric regimen before anti-HCV treatment (6/20 patients, 30%) compared to those who maintained the same psychiatric therapy (11/124, 8.8%) (*p* = 0.015). On the contrary, no differences were observed according to ART change before anti-HCV treatment (no patient failed among those changing ART before baseline while only two failures were evidenced among the remaining subjects).

Excluding lost to follow-up and non compliant patients, according to PP-analysis, SVR-12 was achieved in 93/95 (97.9%) in group A versus 34/36 (94.4%) in group B (*p* = 0.30).

### Safety profile of DAAs

The safety profile was evaluated for the 144 enrolled subjects, and is described in Table 4. Treatment discontinuation was observed in 4 (2.7%) individuals of the study population and were due to: seizures incoming, syncopal episode, severe headache; the fourth patient was detained and interrupted the treatment. A total of 60 patients (41.6%) experienced at least one AE. Most AEs in course of DAAs occurred in patients in treatment with anxiolytics and antidepressant (48.5% vs. 25.6%, *p* = 0.015). The most common AEs were represented by neurological symptoms (18.0%), skin reactions (7.6%), and gastrointestinal disorders (7.6%). Patients receiving RBV more frequently showed AEs during treatment in comparison with subjects treated with RBV-sparing regimens. Erythropoietin use and a RBV dose reduction were reported in 2 (1.3%) and 4 (2.7%) patients, respectively. No statistically significant differences in terms of occurrence of AEs were observed between the two groups. Psychiatric symptoms such as anxiety episodes and mood disorders were reported only among group A patients.

**Table 4:** Safety profile of DAAs regimens

	Patients *(N = 144)*	Group A *(N = 101)*	Group B *(N = 43)*	***p-***value
**At least 1 adverse event,*n*(%)**	60 (41,6%)	49 (48.5%)	11 (25.6%)	**0.015**
**> 2 adverse events,*n*(%)**	7 (4.8%)	6 (5.9%)	1 (2.3%)	0.674
**Severe adverse events,*n*(%)**	10 (6.9%)	8 (7.9%)	2 (4.6%)	0.723
**Adverse events leading to discontinuation,*n*(%)**	3 (2.1%)	2 (1.9%)	1 (2.3%)	1.000
**Skin reactions**^**a**^**,*n*(%)**	11 (7.6%)	9 (8.9%)	2 (4.6%)	0.506
**Anemia,*n*(%)**	7 (4.8%)	5 (4.9%)	2 (4.6%)	1.000
*Requiring erythropoietin*	2 (1.3%)	2 (1.9%)	0	
*Requiring RBV dose adjustment*	4 (2.7%)	2 (1.9%)	2 (4.6%)	
*Requiring hospitalization*	1 (0.7%)	1 (0.9%)	0	
**Gastrointestinal toxicity**^**b**^**,*n*(%)**	11 (7.6%)	9 (8.9%)	2 (4.6%)	0.506
**Cardiac disorders**^**c**^**,*n*(%)**	6 (4.1%)	5 (4.9%)	1 (2.3%)	0.669
**Neurological symtomps,*n*(%)**	26 (18.0%)	20 (19.8%)	6 (13.9%)	0.483
*Asthenia*	15 (10.4%)	12 (11.8%)	3 (6.9%)	0.553
*Headache*	10 (6.9%)	9 (8.9%)	1 (2.3%)	0.281
*Insomnia*	8 (5.5%)	6 (5.9%)	2 (4.6%)	1.000
*Amnesia*	1 (0.7%)	1 (0.9%)	0	1.000
*Seizures*	1 (0.7%)	0	1 (2.4%)	0.298
**Psychiatric symtomps,*n*(%)**	8 (5.5%)	8 (7.9%)	0	0.105
*Anxiety*	1 (0.7%)	1 (0.9%)	0	1.000
*Mood disorders*	7 (4.8%)	7 (6.9%)	0	0.103

*Legend:*^a^-rash, pruritus, photosensitivity;

^b^- diarrhea/constipation, dyspepsia, nausea;

^c^-hyper/hypotension, arrhythmias

Severe AEs were generally uncommon (6.9%). The most common SAE was severe anaemia in 3 patients (2.1%). Three hospitalizations (not leading to treatment discontinuation) were reported due to: angina episode, hyperammonaemic encephalopathy and hematemesis. No death was reported.

## Discussion

The treatment of chronic hepatitis C virus infection has been revolutionized thanks to the recent development of the new oral direct-acting antiviral agents. DAAs increase the likelihood of cure with a shorter duration of treatment and a better safety profile compared to previously used IFN-based regimens.

Despite these overwhelming advances, challenges remain in eliminating HCV in some patient subgroups, such as subjects with decompensated cirrhosis, severe kidney disease, and in the elderly [18]. In addition, among these vulnerable populations, also patients who have psychiatric disorders should be taken into account, for whom IFN-treatment was eluded in previous years or discontinued for severe long-term and incapacitating neuropsychiatric side effects [9]. A certain reluctance of physicians to treat this “special group” can be attributed to concerns about poor treatment adherence or pessimism regarding HCV-treatment tolerability and/or effectiveness even in the DAA era [19]. For all the above mentioned reasons, nowadays these patients are still lacking or delaying access to treatment and cure.

The coexistence of HCV-infection with cognitive disorders is well known, based on studies demonstrating the entrance of HCV in CNS as well as the higher exposure to HCV-infection in patients with psychiatric comorbidities due to more frequent risk behaviors compared to the general population [20]. However, if depression and/or anxiety have been reported in about a third of HCV-infected patients according to different studies [21–23], the prevalence of psychotic disorders (such as schizophrenia, delirious disorder) among HCV infected subjects is not well established, but is estimated about 4% [24]. In our analysis, the overall proportion of HCV-infected patients with psychiatric comorbidity who underwent antiviral therapy was lower than in the above mentioned studies (about 12%), even if restricted inclusion criteria were used, as only patients under a psychiatric treatment were considered eligible.

Our study confirms findings showing that HCV-patients with psychiatric co-morbidity can be treated with DAAs with high efficacy. The rate of SVR-12 obtained in our study (88.2% in ITT-analysis) was consistent with the recent results highlighted from Sundberg et al [25] in whose study 17 patients, despite severe psychiatric morbidity, successfully completed DAA-treatment with a comparable SVR (88%), without worsening psychiatric symptoms. Only few data are available on SVR rates in patients with psychiatric comorbidites [13, 16]. These SVR rates appear only slightly lower compared to those currently reported in the general HCV-infected population, also in our real life experience [26], while it seems to be lower in intravenous drug addicted [14]; We tried to further investigate reasons underlying this gap and for these purposes the population was divided into two groups, based on clinical and therapeutic resemblance, including patients treated with anxiolytics and/or antidepressant in group A, and patients treated with antipsychotics in group B and we find that psychotic patients presented lower SVR rates. It should be beforehand underlined that the two groups were not homogeneous. A first imbalance pertains to the different number of patients in the two groups, with more than twice subjects in group A. A higher percentage of former IDUs was observed in the group of psychotic patients and the population of HCV/HIV-coinfected patients was more represented. As a consequence, differences in the genotype distribution were evidenced: in fact, as expected, genotype 1a and 3 were found widely in group B patients. In contrast, patients in treatment with anxiolytics/antidepressants were significantly older than those in group B, and consequently presented a higher FIB-4 score at baseline, a higher prevalence of cirrhosis, and a higher number of subjects previously treated with unsuccessful IFN-based regimens, probably due to a high rate of IFN-discontinuations. Moreover, a higher number of concomitant comorbidities (hypertension, diabetes, thyreopathies) was reported in patients with anxiety/depression, even if differences between the two groups were not statistically significant. Considered all these differences, it is interesting to highlight that, although group A patients had more hepatic and non-hepatic disabilities and a greater fragility, a lower SVR rate (79%) was observed in psychotic patients compared to others (92%) which was mostly due to a higher proportion of patients lost to follow-up. This could be expected considering some particular aspects characterizing psychotic disorders, probably not completely compensated by the antipsychotic treatment (disorganized behavior, impaired ability of sustained attention). In fact, when considering PP-analysis, a higher SVR-12 rate was observed (97.9% in group A and 94.4% in group B), and our results were in line with recent studies [13, 15, 16]. Morevoer, in a sub-analysis of study population the lack of achievement of SVR-12 was especially reported in former/active intravenous drug use. Finally, we cannot ruled out the possibility that, although patients received an appropriate treatment regimen, the highest proportion of HCV genotype 3 contributed to the lower response in group B, in which was also described the unique relapse (Y93H, NS5A at treatment failure within 12 weeks after EOT).

Moreover, one of our main concerns before starting DAAs and during the course of treatment was the need of an adjustment or modification of the baseline psychiatric co-medication, which was prescribed in over 13.8% of our patients, in agreement with psychiatrists. In fact, changes in psychiatric treatment before DAA-initiation seemed to play a role in treatment failure as it was associated with a higher number of lost to follow-up patients. The lack of a self-rated questionnaire evaluated after the completion of anti-HCV treatment failed to prove whether lost to follow-up patients was due to an underlying psychiatric disorder or other causes (poor medical awareness, doctor-patient relationship). Mostly, psychiatric drugs modifications were adopted among patients in treatment with antipsychotics (up to 19% of cases); the majority of DAAs and antipsychotics shared a same major hepatic landmark in which both were CYP activators or substrates, leading to an increased/decreased exposure to the other drug. Fortunately, the wide availability of new DAA-regimens has nowadays reduced the need of mandatory therapeutic changes. Therefore, our study suggests that in these cases caution is warranted, considering the risk of altering a previous stable mental condition, and a long period of patient monitoring before starting DAAs is desirable. On the contrary, changes should be avoided if not strictly required.

Overall, DAA-based treatment was safe. Almost half of patients (41.6%) experienced at least one mild-to-moderate adverse event. In general, no significant difference was observed in the occurrence and number of AEs between the two groups. As expected, RBV (used in comparable proportion between the two groups) was associated with more AEs in the course of treatment in comparison with patients treated with RBV-sparing regimens. Severe AEs occurred in only 10 patients (6.9%). However, SAEs were less frequent than expected, probably due to the fact that the vast majority of patients had a compensated liver disease. Among 4 individuals who did not achieve EOT, 3/4 reported a treatment discontinuation due to SAEs, including seizure, intense headache, angina pectoris. Nevertheless, the few patients who experienced psychiatric symptoms such as depressive disorder and/or isolated generalized anxiety episode during DAA-treatment were exclusively among subjects in group A whereas none of the patients receiving antipsychotics showed the same disorders. Probably this interesting evidence might be explained by a major aptitude of these patients to perceive the initiation of a new treatment course as a stressing factor [27]. Usually, these symptoms were reported within the first month of treatment.

In a recent review published by Forton et al. [28] the HCV-clearance led to a valid improvement of neuropsychiatric manifestations. In disagreement with this study, in a recent Spanish study DAA-treatment had no impact on anxiety or depression during or after chronic hepatitis C infection treatment, even in high-risk patients with major psychiatric disorders [29]. We cannot confirm this findings as no post-treatment neurospychiatric evaluation was performed; however, in our experience, none of the psychiatric disorders reported during treatment had worsened at the end of treatment.

As widely demonstrated in literature, no difference in efficacy and safety rates were observed in HCV/HIV-coinfected patients compared to HCV-monoinfected. Even if the management of DDIs could be more complicated in these patients, for whom also antiretroviral therapy should be considered, changing ART before baseline did not affect therapy failure in our patients.

The main limitation of our study is its observational, retrospective nature; therefore, some data were not available for the totality of subjects; another limit is the restricted follow-up period after treatment and the lacking of a clinical follow-up based on questionnaires to assess self-reported outcomes.

## Conclusion

Our study points out the complexity of the anti-HCV treatment of HCV-infected patients with psychiatric comorbidity and suggests that slightly lower SVR rates can be expected in psychotic patients while adverse events were more frequently reported among anxious/depressive patients. The study underlines as a careful evaluation of history and all possible drug-drug interactions before starting therapy can have a remarkable impact on the patient outcome, which was overall successful in our experience, thus encouraging a widespread use of DAAs also in such a “special population”.

## Data Availability

The dataset analyzed during the current study is available from the corresponding author on reasonable request.

## Abbreviations

AEs: Adverse events
ART: Antiretroviral therapy
CNS: Central Nervous System
CTCAE: Common Terminology Criteria for Adverse Events
CYP: Cytochrome
DAAs: Direct-acting Antiviral Agents
DCV: Daclatasvir
DDIs: Drug-drug interactions
DSV: Dasabuvir
EBR: Elbasvir
EMA: European Medicines Agency
EOT: End of treatment
FDA: Food and Drug Administration
GLE: Glecaprevir
GRZ: Grazoprevir
HCV: Hepatitis C virus
HIV: Human Immunodeficiency Virus
IDU: Injecting drug user
IFN: Interferon
IQR: Interquartile range
ITT: Intention-to-treat
LDV: Ledipasvir
MSM: Man who have sex with man
OBV: Ombitasvir
PIB: Pibrentasvir
PP: Per-protocol
PTV/r: Paritaprevir/ritonavir
RBV: Ribavirin
SAEs: Severe Adverse events
SD: Standard Deviation
SMV: Simeprevir
SOF: Sofosbuvir
SVR: Sustained Virological Response
VEL: Velpatasvir
VOX: Voxilaprevir
